# Tobacco smoking and the British doctors' cohort

**DOI:** 10.1038/sj.bjc.6602361

**Published:** 2005-02-08

**Authors:** P Boyle

**Affiliations:** 1 International Agency for Research on Cancer, 150 cours Albert Thomas, 69372 Lyon Cedex 08, France

In this issue of the *BJC*, Sir Richard Doll and colleagues from Oxford present findings from the 50 years of follow-up of British doctors in relation to cancer risk (Doll *et al*, 2005). There are many important aspects surrounding this article, some of which deserve wider and deep reflection.

This has been a study that was completely innovative and ingenious in its construction and remarkable in the perseverance of its follow-up. When Richard Doll and Austin Bradford-Hill undertook this cohort study, they probably did not realise that they were setting a new paradigm for modern epidemiology, and choosing to do such a study among doctors was quite ingenious. Which group would be able to be followed to death by a variety of sources including via the Medical Register?

The initial results (Doll and Hill, 1954; Doll and Peto, 1976) were highly significant and of great value in identifying a new and significant cancer risk, but the true worth of this study increased as follow-up increased and the flow of new information emerged. During the course of the follow-up, and in particular in the reports after 40 years follow-up (Doll *et al*, 1994) and 50 years of follow-up (Doll *et al*, 2004), the real impact of tobacco smoking on a wide variety of diseases and life expectancy itself was fully revealed. Half of the smokers die from a tobacco-related disease and half of these deaths occur in middle age. The impact of these deaths on the loss of nonsmokers life expectancy is enormous. Stopping smoking at any age is effective in reducing the loss of nonsmokers life expectancy, although this lessens off as age at quitting increases.

The comparison presented here (Doll *et al*, 2005) is in many respects unique. Here, we have the opportunity to observe what really happens over a long period among those who are exposed to a carcinogenic risk and to compare it with a descriptive analysis of all the available published, epidemiological and mechanistic evidence.

The International Agency for Research on Cancer (IARC) prepared a Monograph on Tobacco Smoking initially in 1986 (International Agency for Research on Cancer, 1986). When this was recently revised (International Agency for Research on Cancer, 2004a) in 2004, there was new information available to increase the numbers of cancer types deemed to be causally related to tobacco smoking (Figure 1). In 11 of 13 cancer types considered by IARC to be causally related to tobacco smoking, and which could be identified on death certificates, Doll and colleagues found them to be significantly related to smoking (Doll *et al*, 2005). For the two remaining sites (nasopharynx, nose and nasal cavity), deaths in the doctors' cohort were sparse, although there was a suggestion that there could well be an association (Doll *et al*, 2005).

In the IARC Monograph (2004a), colorectal cancer and prostate cancer are the two types of cancer that fell between the two classes of *sufficient evidence of carcinogenicity* and *evidence suggesting lack of carcinogenicity*. For colorectal cancer, the Working Group considered that bias and confounding could not be ruled out as alternative explanations of the associations seen, while for prostate cancer, it was felt that the available studies were not mutually consistent in showing a positive association. Doll *et al* (2005) found a potential association restricted to a subgroup of colorectal cancers and no association for prostate cancer. Importantly, there is little evidence of increased site-specific cancer risk for those forms of cancer considered by the IARC Working Group not to be associated with tobacco smoking.

These findings are an excellent example of the robustness of the procedure which the IARC Monographs programme employs to evaluate carcinogenicity of a chemical, biological, physical or lifestyle exposure (Cogliano *et al*, 2004a). An International Working Group that includes the best experts in the field, not tainted by conflicts of interest, reaches their conclusion after thoroughly reviewing all the published database.

The cancer sites identified as causally associated with tobacco smoking in the previous Monograph on tobacco smoking almost 20 years ago (International Agency for Research on Cancer, 1986) were all confirmed in IARC's (2004a) re-evaluation. As many more studies were available for the current evaluation, several cancer sites were added to the list of tobacco-associated cancers (Figure 1).

IARC has now just completed within the Monographs programme a re-examination of all main forms of tobacco, and all of them have been clearly shown to be carcinogenic to humans: tobacco smoking (Figure 1) and involuntary smoking (lung) and the use of betel quid with tobacco (oral cavity, pharynx and oesophagus) (International Agency for Research on Cancer, 2004b) as well as the use of smokeless tobacco (oral and pancreatic cancer) (Cogliano *et al*, 2004b).

Tobacco is the best-identified human carcinogen and is carcinogenic in all its forms of use. It is clear, and has been for several years now, that the effect of tobacco on cancer risk, and indeed on overall mortality, is far in excess of any other common risk factor or treatment effect. Information nowadays taken for granted (half of smokers die of a smoking-related disease, half of these deaths are in middle age, each smoking-related death in middle age loses over 20 years of a nonsmokers life expectancy, there are over 20 fatal diseases causally linked to cigarette smoking, even if a smoker stops smoking in middle age he starts to win back some of nonsmokers life expectancy) has evolved in large part from the work of Sir Richard Doll and his colleagues (in particular Sir Richard Peto) and from the extensive follow-up of the British doctors' cohort.

The early findings from Doll's group (Doll and Hill, 1952, 1954), which clearly identified smoking as a human carcinogen, had a large influence in the great decline in the prevalence of cigarette smoking, which took place in the United Kingdom since the 1950s, and in the United States and many other countries shortly thereafter (Peto *et al*, 2000). This has undoubtedly postponed many deaths in the United Kingdom and in many other parts of the world and has led to millions of men (and women) having several years of increased life expectancy. While such a contribution from any one research group is outstanding, that this group has made major contributions in other major disease areas including radiation and cancer, asbestos and other occupational carcinogens, oral contraceptives and disease, treatment of early breast cancer, immediate treatment of myocardial infarction and aspirin and myocardial infarction is unique and remarkable.

Unsurprisingly, Sir Richard Doll and Sir Richard Peto have received many awards and widespread recognition for their contribution to public health. Such recognition is well deserved even though such statistics-based contributions may well be undervalued (Breslow, 2003). Apart from DA Henderson (who directed the World Health Organization's global smallpox eradication campaign (1966–1977) and helped to initiate WHO's global programme of immunisation in 1974), it is difficult to identify a greater contribution to public health in recent times. They really made a difference.

## Figures and Tables

**Figure 1:**
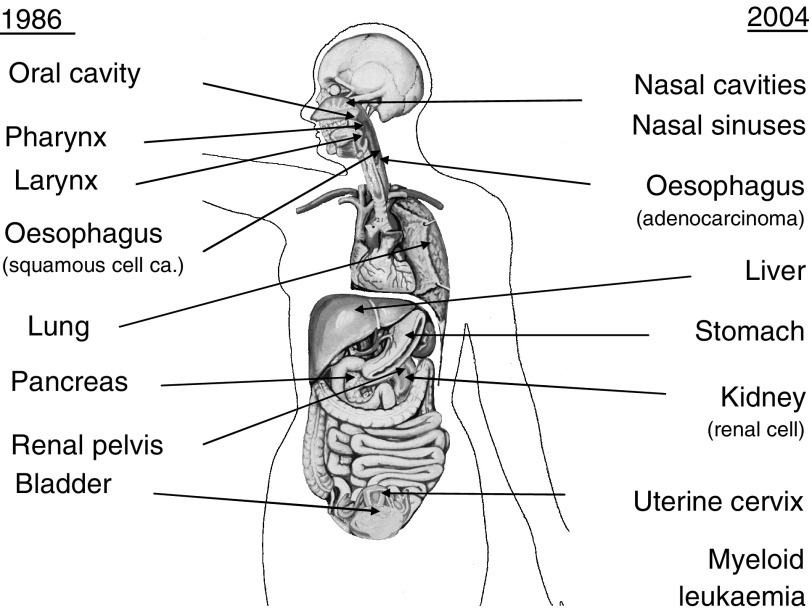
Types of cancer considered to be causally related to tobacco smoking in successive IARC monograph evaluations (International Agency for Research on Cancer, 1986, 2004a).

## References

[bib1] Breslow NE (2003) Are statistical contributions to medicine undervalued? Biometrics 59: 1–8 12762435 10.1111/1541-0420.00001

[bib2] Cogliano VC, Baan RB, Straif K, Grosse Y, Secretan MB, El-Ghissassi F, Kleihues P (2004a) The science and practice of carcinogen identification and evaluation. Environ Health Perspect 112: 1269–1274 15345338 10.1289/ehp.6950 PMC1247515

[bib3] Cogliano V, Straif K, Baan R, Grosse Y, Secretan B, El Ghissassi F (2004b) Smokeless tobacco and related nitrosamines. Lancet Oncol 5: 708 15600001 10.1016/s1470-2045(04)01633-x

[bib4] Doll R, Hill AB (1952) A study of the aetiology of carcinoma of the lung. BMJ 2: 1271–1286 12997741 10.1136/bmj.2.4797.1271 PMC2022425

[bib5] Doll R, Hill AB (1954) The mortality of doctors in relation to their smoking habits: a preliminary report. BMJ 228: 1451–1455 10.1136/bmj.1.4877.1451 PMC2085438 13160495

[bib6] Doll R, Peto R (1976) Mortality in relation to smoking: 20 years observations on male British doctors. BMJ 2: 1525–1536 1009386 10.1136/bmj.2.6051.1525 PMC1690096

[bib7] Doll R, Peto R, Wheatley K, Gray R, Sutherland I (1994) Mortality in relation to smoking: 40 years' observations on male British doctors. BMJ 309: 901–911 7755693 10.1136/bmj.309.6959.901 PMC2541142

[bib8] Doll R, Peto R, Boreham J, Sutherland I (2004) Mortality in relation to smoking: 50 years' observations on male British doctors. BMJ 328: 1519–1528 15213107 10.1136/bmj.38142.554479.AE PMC437139

[bib9] Doll R, Peto R, Boreham J, Sutherland I (2005) Mortality from cancer in relation to smoking: 50 years' observations on British doctors. Br J Cancer 92, (this issue) 10.1038/sj.bjc.6602359 PMC2362086 15668706

[bib10] International Agency for Research on Cancer (IARC) (1986) Monographs on the Evaluation of Carcinogenic Risks to Humans Volume 38. Tobacco Smoking. Lyon: IARC

[bib11] International Agency for Research on Cancer (IARC) (2004a) Monographs on the Evaluation of Carcinogenic Risks to Humans Volume 83. Tobacco Smoke and Involuntary Smoking. Lyon: IARC PMC4781536 15285078

[bib12] International Agency for Research on Cancer (IARC) (2004b) Monographs on the Evaluation of Carcinogenic Risks to Humans Volume 85. Betel-quid and Areca-nut Chewing and Some Areca-nut derived Nitrosamines. Lyon: IARC PMC4781453 15635762

[bib13] Peto R, Darby S, Deo H, Silcocks P, Whitley E, Doll R (2000) Smoking, smoking cessation and lung cancer in the UK since 1950: combination of national statistics with two case–control studies. BMJ 321: 323–329 10926586 10.1136/bmj.321.7257.323 PMC27446

